# The First and Final Answer: Left Internal Thoracic Artery-to-Left Anterior Descending Artery Bypass and the Reappraisal of Coronary Revascularization

**DOI:** 10.3390/jcm15124813

**Published:** 2026-06-21

**Authors:** Katsuhiko Oda, Makoto Takahashi, Ryuichi Taketomi, Kota Itagaki, Takehiro Sato, Shintaro Katahira

**Affiliations:** Department of Cardiovascular Surgery, Iwate Prefectural Central Hospital, Morioka 020-0066, Japan; popeyethesailormoonjupiter@yahoo.co.jp (M.T.); ryuichi.taketomi@gmail.com (R.T.); kota.itagaki.e6@tohoku.ac.jp (K.I.); seika-tennis-medaka@docomo.ne.jp (T.S.); shinta0911@yahoo.co.jp (S.K.)

**Keywords:** ischemic heart disease, coronary artery bypass grafting, left internal thoracic artery, left anterior descending artery, medical therapy, percutaneous coronary intervention, coronary revascularization, total graft flow plateau, complete revascularization, myocardial perfusion

## Abstract

Medical intervention for ischemic heart disease began approximately 150 years ago with nitrates, and for nearly a century thereafter, little fundamental progress was made. With the advent of the left internal thoracic artery to the left anterior descending artery (LITA-LAD) bypass in the 1960s, treatment entered a new stage; however, its essential significance remained insufficiently recognized for many years. Numerous studies were subsequently conducted to evaluate alternative or parallel treatment strategies, but these investigations also helped bring the durable capacity of LITA-LAD to perfuse ischemic myocardium into sharper focus. Over the past quarter century, the treatment of ischemic heart disease has remained in a state of uncertainty, and its central prognostic foundation has often been obscured, although in recent years this uncertainty has begun to resolve. In this review, we reexamine the historical process by which the significance of LITA-LAD remained incompletely appreciated in parts of the cardiology and cardiac surgical communities. We further outline how the principal basis of the long-term prognostic benefit conferred by LITA-LAD gradually became evident and define the contemporary roles of medical therapy and percutaneous coronary intervention in relation to LITA-LAD-based coronary artery bypass grafting (CABG).

## 1. Introduction

The history of medicine is not a linear process in which rational and appropriate therapies are steadily developed and widely adopted. At times, treatments of uncertain value become widely adopted, while valuable therapies remain underrecognized, challenged, or obscured. Ischemic heart disease provides a clear example of this pattern. CABG was not immune to this tendency. Although its history is often presented as a progressive refinement of surgical revascularization, a central historical feature of CABG is that its foundational solution had already appeared at the very beginning. Subsequent decades of research formally compared competing strategies, conduits, and technologies, yet much of that literature was built upon a clinical foundation already transformed by left internal thoracic artery to left anterior descending artery (LITA-LAD) bypass. In this sense, the history of CABG may be understood as the delayed recognition of a solution that had been achieved at the outset.

In contrast to operations in which prosthetic devices or artificial materials often represent the principal technical variable, CABG is inherently dependent on the patient’s own vessels. Because of this characteristic, the feasibility and selection of arterial grafts may reflect the patient’s underlying condition, anatomy, and operative risk. Consequently, retrospective comparisons of conduits are particularly vulnerable to selection bias. Since randomized controlled trials (RCTs) provide a higher level of evidence than retrospective studies, the present review places primary emphasis on RCT evidence.

This review reconstructs the history of CABG not as a parallel competition among multiple strategies, conduits, and technologies, but as a process in which the foundational solution—LITA-LAD—emerged early, while its full significance remained unrecognized for a prolonged period. Viewed from this perspective, the history of CABG can be understood as a prolonged process of evaluation and reinterpretation that ultimately refocused attention on LITA-LAD bypass, which had already emerged in the 1960s.

This article is intended as a narrative and historical-conceptual review written from a surgical expert perspective, rather than as a systematic review. It integrates early historical reports, landmark randomized trials, long-term follow-up studies, guideline-relevant evidence, and selected technical and physiological observations related to LITA-LAD, CABG, PCI, medical therapy, conduit selection, and complete revascularization to reinterpret the development of coronary revascularization around LITA-LAD. No formal search strategy or predefined inclusion and exclusion criteria were applied; therefore, the literature selection was non-systematic and reflects an interpretive synthesis by the authors. The literature considered spans the early development of surgical myocardial revascularization in the mid-twentieth century through contemporary evidence available at the time of manuscript preparation. Within this scope, contemporary clinical relevance is considered briefly, particularly in settings in which LITA-LAD-based CABG remains relevant when surgical revascularization is selected. The phrase “first and final answer” is used as a conceptual framing rather than as a simplified chronological priority claim: LITA-LAD appeared early in the development of CABG and later emerged as the durable surgical basis for long-term prognostic benefit.

## 2. The Dawn of Ischemic Heart Disease Treatment: From Nitrates to the Eve of LITA-LAD

### 2.1. The Era of Symptomatic Relief

Ischemic heart disease, presenting as chest pain, became an early target of medicine’s most fundamental aim: the relief of suffering. Accordingly, treatment began not with the correction of ischemia but with symptom relief. In the late nineteenth century, antianginal therapy entered a new phase in Britain. Amyl nitrite was introduced first, but nitroglycerin soon became the principal antianginal agent [1]. Its emergence was historically notable: the same compound had become central to modern explosives through Nobel’s dynamite, which underpinned mining, tunneling, and infrastructure development in the industrial age, while in medicine it offered one of the first effective means of relieving angina. Importantly, its clinical success preceded a full understanding of its mechanism by nearly a century, until nitric oxide was recognized as central to its mode of action [2]. Yet the heart remained beyond the reach of effective surgical treatment. As a result, symptomatic therapy spread worldwide and dominated the treatment of ischemic heart disease for nearly a century.

### 2.2. The Birth of Qualitative Diagnosis

A major step in the history of ischemic heart disease was the gradual emergence of qualitative diagnosis. Caleb Hillier Parry had linked angina pectoris to coronary artery disease, thereby moving the condition beyond a symptom complex toward a pathophysiologic entity [3]. The twentieth century transformed this insight into clinical reality. With Willem Einthoven’s invention of electrocardiography (ECG) in 1901 [4], ischemic myocardial injury gradually became recognizable in living patients rather than only inferred from symptoms or autopsy findings. By 1920, ECG findings associated with myocardial infarction had already been described [5]. Because ECG did not require large-scale infrastructure and could be applied relatively widely, it was particularly well suited for global dissemination. Yet direct treatment remained beyond reach, and management continued to rely largely on nitrates.

### 2.3. Toward Surgical Treatment: Vineberg, Sones, and the Eve of LITA-LAD

The two World Wars likely imposed substantial physical and psychological stress on populations and, together with broader social changes, contributed to the rise in ischemic heart disease [6,7]. At the same time, the wars substantially increased the operative experience of military surgeons: through repeated exposure to thoracic trauma and cardiac or great-vessel injury, the longstanding notion of the heart as an untouchable organ began to erode. By the first half of the twentieth century, an atmosphere had emerged in which a treatment beyond symptomatic relief with nitrates was increasingly desired. It was in this historical setting that Arthur Vineberg began experimental implantation of the LITA into the myocardium in 1946 and performed the first clinical operation in 1950 [8]. From a modern perspective, the procedure was clearly indirect: LITA mobilization and intramyocardial implantation without direct coronary anastomosis. Yet in some patients anginal symptoms improved, suggesting a possible physiological effect. Because the results were still incomplete and no better alternative existed, the operation persisted despite considerable skepticism. In 1958, however, Mason Sones introduced selective coronary angiography (CAG), opening an era in which coronary artery disease itself could finally be visualized directly in living patients [9]. CAG did not immediately solve the therapeutic problem, but it substantially changed the conceptual basis for surgical treatment.

## 3. The Birth of LITA-LAD: The Earliest and Foundational Form of Coronary Revascularization

### 3.1. Recognition of the LITA as a Suitable Arterial Conduit

Vineberg surgery likely prompted cardiac surgeons to reflect on the nature of the LITA, drawing attention to its anatomical characteristics and physiological potential. Vineberg himself also recognized that the LITA was a vessel relatively spared from atherosclerotic disease, a feature that would have made it particularly attractive even before the era of direct coronary bypass [10]. At that time, cardiac surgery was still in its infancy, and many cardiac surgeons had broad experience in general and vascular surgery. It is therefore plausible that they could recognize the LITA as a potential collateral pathway to the lower extremities through the inferior epigastric artery, particularly in patients with lower limb ischemia, in whom this pathway could occasionally become markedly developed [11].

### 3.2. Coronary Angiography and the New Map of Ischemic Heart Disease

In contrast, Sones’s selective CAG provided direct visualization of the coronary circulation, including the left main trunk, its bifurcation into the left anterior descending (LAD) and circumflex arteries (LCx), the right coronary artery (RCA), and their major branches [12]. It also revealed the size and morphology of these vessels, substantial interindividual anatomical variation, and, in cases of occlusion, collateral supply from other coronary arteries. It can therefore be inferred that CAG was recognized early as a method that provided clinically important information for planning the treatment of ischemic heart disease. In addition, because CAG did not require particularly large-scale equipment, it was well suited for wide dissemination. Unlike valvular heart disease, for which echocardiography became indispensable, or aortic disease, for which contrast-enhanced computed tomography became essential, ischemic heart disease may be regarded as a field that reached a diagnostic environment broadly comparable to that of the modern era at a relatively early stage.

### 3.3. Technical Preconditions for Direct Coronary Anastomosis

Cardiopulmonary bypass (CPB) was developed in the 1950s [13], but the technology and its adjuncts were still in an early stage of development. Nevertheless, because the coronary arteries lie on the surface of the heart, coronary reconstruction did not necessarily require complete cardiac arrest and, under certain circumstances, was not technically impossible even without the use of CPB. In practice, however, one major obstacle had to be overcome: how to create a high-quality anastomosis in a 1–2 mm coronary artery on the beating heart, and how to ensure the reliability of that anastomosis. In addition, myocardial protection was still developing in this era, and extracorporeal circulation had not yet reached the degree of refinement seen in later decades; prolonged cardiac arrest and prolonged CPB time were therefore not conditions associated with favorable results. Yet coronary anastomosis itself did not necessarily require a long period of time. In this sense, coronary surgery may be regarded as a field that remained technically achievable even in the era of still-developing CPB.

Furthermore, fine surgical instruments such as the specialized needle holder developed by Castroviejo for ophthalmic surgery were already beginning to extend beyond their original field [14]. Although the widespread adoption of monofilament nonabsorbable sutures such as polypropylene would come somewhat later, fine nylon and silk sutures were already available, meaning that many of the basic technical prerequisites for coronary anastomosis were in place. In addition, unlike valvular surgery, which required the development of prosthetic valves, or aortic surgery, which depended on prosthetic vascular grafts, coronary surgery did not necessarily require elaborate medical materials whose development demanded substantial time and cost. The patient’s own vessels could be used. In this respect, the field held a practical advantage. Viewed in this light, direct coronary anastomosis had already reached the stage at which it was no longer only a conceptual possibility; what remained was the technical execution of the anastomosis itself.

### 3.4. Kolesov and the First Sutured Direct ITA-Coronary Anastomosis

An earlier internal thoracic artery (ITA)-coronary artery anastomosis had been performed by Robert H. Goetz in 1960 using a nonsuture mechanical device [15]. However, the first successful sutured direct ITA-coronary artery anastomosis was achieved by Vasilii I. Kolesov, a cardiac surgeon in Leningrad, then in the Soviet Union [16]. On 25 February 1964, he performed an anastomosis between the LITA and the LCx on the beating heart, establishing the first sutured direct ITA-coronary artery anastomosis. Kolesov subsequently extended this method to the LITA-LAD anastomosis as well, thereby anticipating the configuration that would later become the standard form of CABG. Yet the historical significance of his work was not immediately recognized internationally, despite the publication of his clinical experience in a major American surgical journal. Rather, its pioneering importance became widely appreciated only after Olearchyk’s 1988 review [17].

## 4. The Completion of LITA-LAD and the Headwinds It Faced

### 4.1. Kolesov and Green: Recognition of the Foundational Form of Coronary Revascularization

By the mid-1960s, the foundational concept of LITA-LAD had already been established. Kolesov showed that the LITA could be directly anastomosed to the LAD and provide durable clinical benefit, while George E. Green independently recognized in the United States that direct LITA-LAD anastomosis represented the durable surgical solution for coronary revascularization [18]. In retrospect, Kolesov and Green were among the earliest surgeons to recognize that LITA-LAD was the foundational form of coronary revascularization.

### 4.2. Early Rejection in the Soviet Union and the United States

The resistance that followed is historically important because it reveals how direct LITA-LAD anastomosis was initially perceived. Kolesov’s work was not widely accepted even in the Soviet Union, where, in 1967, the plenum of the All-Union Cardiological Society adopted a resolution declaring that surgical treatment of coronary artery disease was impossible and without prospects [17]. American skepticism was also visible in the editorial handling of Kolesov’s 1967 report [16]: the editor explicitly noted that his views were at variance with the concept of many surgeons in the United States and therefore invited Donald B. Effler to provide a comment. Green’s experimental success did not lead to immediate clinical acceptance at New York University [10]. Adrian Kantrowitz found the data interesting but requested that the experiments be repeated in his own laboratory before any clinical translation was attempted. Donald B. Effler was impressed by the concept yet still framed the procedure as something Green should pursue only under his supervision. Michael E. DeBakey also rejected clinical application, stating that coronary bypass surgery was not feasible.

### 4.3. Hesitation, Diversion, and the Persistence of Indirect Revascularization

This skepticism did not take only the form of outright opposition. Even after bypass surgery began to rise, René G. Favaloro still acknowledged a role for the Vineberg operation and recognized the considerable technical difficulty of LITA anastomosis, particularly because of the fragile wall of the LITA. Similarly, rather than adopting direct LITA grafting, Alain F. Carpentier introduced the radial artery in 1973 as a technically more accessible arterial alternative; however, early RA grafting proved disappointing and was largely abandoned [10]. More broadly, many surgeons continued to work within the framework of indirect revascularization, and this persistence delayed full recognition of direct LITA-LAD grafting. Thus, the early history of LITA-LAD was not one of rapid acceptance but one of repeated resistance, hesitation, and diversion. The operation that would later become the cornerstone of CABG was, at its birth, perceived by many leading surgeons as technically unrealistic, clinically premature, or not yet superior to the indirect or alternative approaches then available.

### 4.4. Technical Difficulty, Not Conceptual Weakness

These reactions did not indicate conceptual weakness. Rather, they showed that LITA-LAD was a highly demanding operation. It required delicate harvesting of a fragile conduit, precise distal LAD target selection, and refined small-vessel anastomotic skill. Thus, what spread first was not necessarily the most durable operation, but the technically less demanding one. The graft with a highly favorable combination of long-term patency, growth potential, and ischemia-relieving capacity was identified early but recognized late.

### 4.5. From Technical Depth to Symbolic Simplification

Paradoxically, once the clinical value of LITA-LAD became widely accepted, its technical depth became less visible behind the label itself. The early responses of several leading cardiac surgeons—ranging from resistance to hesitation or diversion—suggest that the quality of this operation should be understood as dependent on technical execution and surgical skill. Yet “LITA-LAD” came to function as a simple label, as though it described a self-evident and uniformly reproducible maneuver. This shift from early resistance to later symbolic simplification may have contributed to subsequent research designs that treated LITA-LAD as a uniform procedural variable rather than as a technically demanding and skill-dependent operation.

This tendency has not entirely disappeared even in contemporary historical narratives. For example, a recent expert opinion published 60 years after the first CABG framed the early history of CABG primarily in terms of the saphenous vein operation performed by Garrett and DeBakey, while referring only briefly to earlier experimental and limited clinical applications of the internal thoracic artery that had faced skepticism [19]. Such a narrative is historically understandable from the standpoint of the rapid dissemination of GSV-CABG, but it also illustrates how the importance of direct LITA-LAD grafting can remain underemphasized even today. In this sense, the underrepresentation of Kolesov’s and Green’s contributions is not merely a historical curiosity, but also a reminder that the historical and conceptual importance of LITA-LAD has still not been fully incorporated into the standard narrative of CABG.

## 5. Partner or Competitor?

### 5.1. The Rise in GSV-CABG as the First Widely Adopted Competitor to LITA-LAD

The first major competitor to emerge alongside LITA-LAD was CABG using the great saphenous vein (GSV), which was clinically established and popularized by René G. Favaloro [20]. Because GSV grafting required a much lower technical threshold than LITA-LAD anastomosis, it spread rapidly throughout the world. Together with the hesitation of many cardiac surgeons to adopt the technically demanding LITA-LAD grafting, this expansion created the impression that LITA-LAD might remain a specialized technique rather than become a mainstream approach in coronary surgery.

### 5.2. Long-Term Evidence Establishing LITA-LAD Superiority

**Figure 1:**
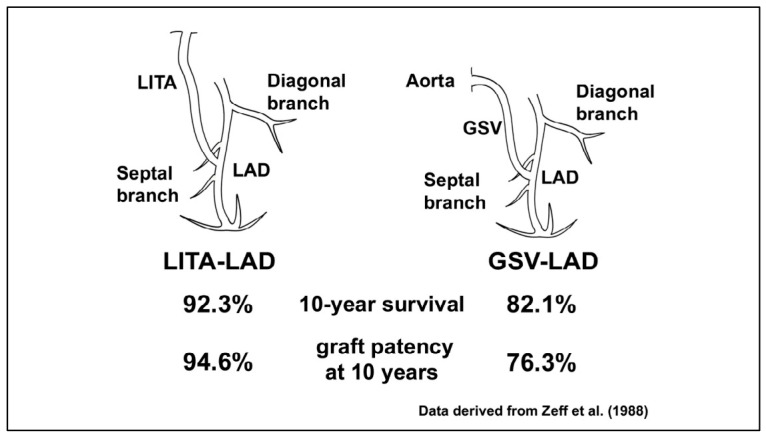
Long-term superiority of LITA-LAD over GSV-LAD. Direct comparison of left internal thoracic artery-to-left anterior descending artery bypass (LITA-LAD) and great saphenous vein-to-left anterior descending artery bypass (GSV-LAD), based on the randomized trial of Zeff et al. [22]. When the target was standardized to the LAD, LITA-LAD showed clear advantages in long-term survival and graft patency. These findings established the long-term advantage of LITA-LAD for surgical revascularization of the LAD territory. GSV, great saphenous vein; LAD, left anterior descending artery; LITA, left internal thoracic artery. This figure is intended as a conceptual illustration of an important historical randomized comparison and should not be interpreted as definitive evidence by itself.

However, in surgery for ischemic heart disease, the clinical durability of revascularization becomes evident only with long-term follow-up. When myocardial revascularization is physiologically or technically inadequate, patients may redevelop symptoms and, in some cases, experience fatal cardiac events. As summarized in Figure 1, with accumulating follow-up, the superiority of LITA-LAD became increasingly evident. Loop’s retrospective study in 1986 [21] and Zeff’s randomized trial in 1988 [22] provided strong support for this recognition. In the LAD territory, the randomized comparison by Zeff et al. showed that LITA-LAD was associated with an approximately 10% advantage in 10-year survival compared with GSV-LAD [22]. This substantial difference between LITA and GSV for LAD revascularization became a fundamental reference point for interpreting the many graft-comparison studies that followed.

### 5.3. Beyond Conduit Selection: The Quality of LAD Revascularization

Zeff’s trial is often interpreted simply as a comparison between LITA and GSV as conduits. However, its broader implication is clinically important: the quality of revascularization of the LAD territory is a determinant of long-term survival. This principle extends beyond conduit selection. Just as a less durable conduit to the LAD compromises survival, a technically suboptimal LITA-LAD anastomosis may also undermine the survival benefit of CABG, regardless of the conduit used. The coronary surgical community has largely embraced LITA-LAD as a label, while the quality of its execution has received comparatively little systematic attention. In this sense, Zeff’s message has not yet been fully incorporated into the interpretation of LITA-LAD-based CABG.

### 5.4. PCI, Medical Therapy, and the Repositioning of CABG

By the late 1980s, when LITA-LAD had become established as the gold standard for LAD revascularization and the GSV had shifted toward the role of the second conduit, percutaneous coronary intervention (PCI), first introduced in 1977 [23], was also entering its own developmental trajectory, progressing from percutaneous transluminal coronary angioplasty (PTCA) to bare-metal stents (BMSs) and later to drug-eluting stents (DESs) [24,25]. Initially, because restenosis was frequent, PCI did not pose a major challenge to CABG. Over time, however, as the technology advanced, PCI gradually emerged as a major competitor. In this setting, a growing concern emerged within the field: could GSV, which had shown inferior long-term performance to LITA for LAD revascularization, remain acceptable as the second graft, or would CABG itself eventually be displaced by PCI if this limitation remained unaddressed?

At the same time, medical therapy was also changing. For many decades, nitrates had served primarily as symptomatic treatment, but this period saw the introduction of aspirin, statins, ACE inhibitors, and other agents supported by clear evidence for secondary prevention in ischemic heart disease [26]. Thus, from the 1980s into the 1990s, CABG was increasingly repositioned in relation to two expanding modalities: PCI, which emerged as a major competitor, and evidence-based medical therapy, which became an increasingly important partner in secondary prevention, while the role of GSV as the second conduit remained unresolved.

### 5.5. PCI as an Essential Therapy in Acute Coronary Syndromes (ACS)

It is important to distinguish the essential role of PCI in acute coronary syndromes from its more limited value in stable ischemic heart disease. In ST-elevation myocardial infarction, primary PCI functions as an emergency reperfusion therapy, and a quantitative review of 23 randomized trials showed better clinical outcomes with primary angioplasty than intravenous thrombolytic therapy [27]. In unstable coronary artery disease, corresponding largely to non-ST-elevation acute coronary syndromes in contemporary terminology, the FRISC II trial showed that an early invasive strategy reduced death or myocardial infarction compared with a non-invasive strategy in patients with evidence of ischemia or myocardial injury [28]. Thus, the clinical value of PCI in ACS is not in question. The difficulty arose when the success of PCI in ACS was extended to stable ischemic heart disease and used to support the broader expectation that PCI might rival or replace CABG as a prognostic therapy.

### 5.6. From Complementary Modalities to a Prolonged Competitive Framework

Underlying these tensions, when considering the treatment of ischemic heart disease, a fundamental question has persisted from the 1980s onward: should therapy restore flow by dilating the stenotic segment itself, as in PCI; should it create an alternative route of blood supply independent of the diseased segment, as in CABG; or should it treat the underlying atherosclerotic process pharmacologically, as in medical therapy? The optimal balance among these approaches has been debated for decades.

In principle, all these therapies were aimed at treating the same disease. They therefore could have been viewed as complementary modalities to be combined according to clinical context for the benefit of the patient. However, a more competitive hypothesis gained influence: that PCI might rival CABG or provide a comprehensive revascularization strategy for ischemic heart disease. Under this assumption, numerous studies involving large numbers of patients were repeatedly designed and conducted. With each new result, the field moved between optimism and reassessment, and clinical guidelines were repeatedly revised, leading to a prolonged period of uncertainty.

## 6. The Resolution of the Second-Graft Controversy

### 6.1. Observational Origins of the Second-Graft Controversy

The first major issue arising from the incomplete recognition of the central role of LITA-LAD was the second-graft controversy in CABG: which conduit should be used once LITA-LAD had been established. This debate was shaped by influential observational reports from Lytle et al. in 1999 and 2004 [29,30], which suggested that, among patients receiving an ITA graft, the use of two ITAs was associated with better long-term outcomes than the use of one. These reports stimulated more than two decades of comparisons among the right internal thoracic artery (RITA), RA [31,32], right gastroepiploic artery (RGEA) [33,34], and GSV. However, because second-conduit selection is strongly influenced by patient characteristics, surgeon preference, institutional practice, and the quality of the index LITA-LAD graft, observational comparisons were limited in their ability to resolve the controversy definitively. Lytle and colleagues themselves acknowledged that such observational comparisons were affected by patient-selection bias and that an RCT would be the ideal method to eliminate it, although they regarded such a trial as difficult to conduct. This limitation was illustrated by the large Mayo Clinic series by Locker et al., in which the apparent survival advantage of multiple arterial grafting was markedly attenuated after propensity-score matching. The authors also acknowledged that missing covariates and selection preferences could have contributed to the better outcomes observed in the multiple-arterial-grafting group [35]. Nevertheless, for many years thereafter, no RCT of bilateral versus single ITA grafting was undertaken in the United States.

### 6.2. The Key Prerequisite: An Equivalent LITA-LAD Foundation

The premise underlying this long-standing controversy is straightforward: once LITA-LAD is established as the foundation of CABG, a second arterial conduit with better long-term patency than GSV should theoretically improve long-term outcomes. However, this premise depends on one important prerequisite: if the principal survival benefit of CABG resides in LITA-LAD, any informative second-graft comparison requires comparable LITA-LAD quality, patency, and functional contribution between groups. Many studies recorded whether LITA-LAD was performed but did not verify whether its quality, patency, or functional contribution was comparable between groups. Therefore, their ability to resolve the second-graft controversy is limited.

### 6.3. Goldman’s CSP 474 Trial: Documented LITA-LAD Patency as the Foundation for Comparison

In this regard, Goldman’s CSP 474 trial is particularly informative [36,37]. In the original randomized report, 1-year graft patency was directly assessed, and LITA-LAD graft patency was similarly high in both the RA and GSV groups [36]. Under these conditions, RA did not demonstrate superiority over GSV as a second graft. Although 1-year patency does not fully capture the long-term functional contribution of LITA-LAD, it showed that the index LITA-LAD graft was comparably patent between groups at an early postoperative stage. The subsequent long-term follow-up of the same randomized trial showed no survival advantage of RA over GSV through nearly 18 years [37]. Thus, Goldman’s CSP 474 trial provides important evidence because it tested the second-conduit hypothesis under conditions in which the index LITA-LAD graft had documented comparable early patency.

### 6.4. ART: A Large-Scale Randomized Comparison of Realized Second-Graft Strategies

In this context, ART provides complementary evidence at a much larger scale [38,39]. Conducted outside the United States by an international group led from Oxford, ART did not directly document the quality, patency, or functional contribution of ITA-LAD, but its randomized design and large sample size of more than 3000 patients likely reduced group-level variation in background ITA-LAD quality. Therefore, ART can be regarded as an informative randomized comparison of realized second-graft strategies built on a common ITA-LAD foundation.

In ART, the realized second-graft distribution was 86.1% second ITA and 13.9% GSV in the BITA-assigned group, and 78.2% GSV and 21.8% RA in the SITA-assigned group. Nevertheless, no difference in 10-year survival was observed between the two assigned groups. Thus, based on this realized conduit use, ART approximated a clinically important comparison: a strategy dominated by second ITA use versus a strategy dominated by GSV use on a common ITA-LAD foundation. If the second graft were a major independent determinant of long-term survival, a difference should have emerged in this setting. No such difference was observed.

### 6.5. Practical Resolution: High-Quality LITA-LAD First, Pragmatic Second-Graft Selection

Taken together, Goldman’s CSP 474 trial and ART indicate that differences in second-graft selection were not associated with a detectable survival advantage on a LITA-LAD foundation. This interpretation is consistent with Subramanian et al. [40], who reported that, in patients with a patent LITA-LAD graft, medical therapy, PCI, and redo CABG for non-LAD ischemia were not associated with differences in survival. In our interpretation, the available randomized evidence indicates a practical resolution of the long-standing second-graft controversy with respect to survival superiority, while not constituting a definitive settlement of all conduit-related questions.

These findings have important implications for future research and surgical practice. ART enrolled more than 3000 patients and showed no 10-year survival advantage of the BITA strategy, despite a higher incidence of sternal wound complications. Goldman’s CSP 474 trial likewise showed no long-term survival advantage of RA over GSV. In contemporary practice, prior trans-radial catheterization may further limit the suitability of RA as a conduit in many patients. After these two major randomized trials, further large-scale trials exposing patients to additional arterial harvesting to demonstrate incremental survival gains would require careful justification, clearly defined target populations, and clinically meaningful endpoints. The practical implication is straightforward: the primary surgical objective should be a high-quality LITA-LAD. The second graft should be selected pragmatically for non-LAD perfusion, while LITA-LAD remains the central focus of CABG.

Why, then, can a LITA-LAD foundation be sufficient to sustain the long-term survival benefit of CABG? Answering this question requires an understanding of the LAD-centered myocardial perfusion system. This question will be revisited in Section 8.

Representative randomized evidence comparing coronary bypass conduits is summarized in Table 1.

## 7. The PCI Challenge: The Absence of a Single Definitive Trial and the Indirect Comparison

### 7.1. The Revival of PCI Enthusiasm Amid Early Uncertainty

The second major controversy concerned PCI versus CABG. PCI evolved rapidly because it offered the possibility of revascularization without sternotomy or surgical graft harvesting. However, as early as 1988, several studies had questioned whether focal dilation of angiographically severe stenoses could fully prevent future myocardial infarction by showing that subsequent myocardial infarction often arose from lesions that had not previously appeared severely stenotic [46,47]. In 1999, the AVERT trial further showed that aggressive statin therapy could achieve outcomes at least comparable to, and in some analyses more favorable than, PCI in selected patients with stable coronary artery disease [48]. Thus, by the end of the twentieth century, the prognostic role of PCI was already uncertain. The SIRIUS trial in 2003 substantially altered this landscape by demonstrating a marked reduction in restenosis with DESs, thereby reviving enthusiasm for PCI as a less invasive revascularization strategy in selected patients [49].

### 7.2. The Absence of a Definitive Trial Comparing PCI with LITA-LAD-Based CABG

As shown in Table 2, however, the PCI literature [50,51,52,53,54,55,56,57,58,59,60,61,62,63,64] did not consistently test PCI against CABG under conditions in which the largest prognostic separation would have been expected: long-term survival in high-risk patients with low-EF ischemic cardiomyopathy, complex three-vessel disease, high-risk left main disease, or severe diabetes mellitus. This structural feature allowed a prolonged period of uncertainty to persist, leaving the relative prognostic roles of PCI and CABG incompletely defined in stable ischemic heart disease, despite limited evidence of long-term prognostic benefit from PCI in this setting.

### 7.3. Repeated PCI Iterations Compared with a Relatively Stable Surgical Comparator

Table 3 further shows that these trials were conducted after LITA-LAD had already become the gold standard of CABG. Across trials, the basic surgical strategy remained largely consistent: LITA-LAD-based surgical revascularization with additional grafting to non-LAD territories. In contrast, PCI was repeatedly evaluated in new clinical trials as its technology evolved from PTCA to BMSs and then to DESs. Each iteration generated new randomized trials, often in selected populations and in some cases with industry involvement. Thus, while PCI technology changed repeatedly, the comparator was largely the same LITA-LAD-based CABG strategy. Although formally randomized, these trials were conducted within preselected PCI-feasible populations, excluding many patients unsuitable for PCI but still amenable to CABG. Thus, they were not designed to provide a definitive comparison across the broader spectrum of high-risk ischemic heart disease. Nevertheless, the persistence of comparable or favorable CABG outcomes even in these selected PCI-feasible populations further supports the durability of outcomes with LITA-LAD-based surgical revascularization.

### 7.4. The Indirect Comparison: PCI Versus Medical Therapy and CABG Versus Medical Therapy

As evidence accumulated, PCI’s role as a prognostic therapy in stable coronary disease became increasingly limited. DESs reduced restenosis, but subsequent trials progressively narrowed the expected prognostic role of PCI in this setting. COURAGE in 2007 [67] did not demonstrate a prognostic advantage of PCI added to contemporary medical therapy in stable coronary disease. FAME in 2009 [71] then highlighted the limitations of angiography-driven PCI by showing that intervention should be restricted to physiologically significant lesions. Thus, FAME did not merely refine PCI; it also called into question the earlier practice of treating angiographic stenosis as a sufficient indication for intervention. ISCHEMIA in 2020 [72] did not demonstrate a prognostic advantage of an initial predominantly PCI-based invasive strategy over contemporary medical therapy, and REVIVED-BCIS2 in 2022 [68] showed that PCI added to medical therapy did not reduce death or hospitalization for heart failure even in ischemic cardiomyopathy with severely reduced left ventricular function. ORBITA in 2018 [73] added a different perspective by showing, in a sham-controlled double-blind trial, that PCI did not significantly increase exercise time beyond the effect of a placebo procedure in patients with medically treated angina and severe coronary stenosis. In contrast, STICHES in 2016 [70] demonstrated that CABG added to medical therapy improved long-term outcomes in patients with ischemic cardiomyopathy and severely reduced left ventricular function.

### 7.5. A Contemporary Framework for Ischemic Heart Disease Treatment

Thus, the roles of the three major strategies have converged toward a more evidence-aligned framework. Contemporary medical therapy forms the basis of treatment for many patients with stable ischemic heart disease. PCI remains important for ACS and selected symptomatic lesions. CABG, particularly LITA-LAD-based surgical revascularization, remains a treatment strategy with demonstrated prognostic value in patients with sufficiently high anatomical complexity, biological risk, or functional risk. In this sense, the period in which PCI adoption was driven by procedural feasibility, technological progress, and expanding clinical use despite limited definitive prognostic evidence in stable disease is now giving way to a more evidence-based understanding of ischemic heart disease treatment.

## 8. A Contemporary CABG Strategy Centered on High-Quality LITA-LAD

### 8.1. Re-Centering CABG on High-Quality LITA-LAD

The preceding chapters suggest that contemporary CABG should be re-centered on the quality and physiological role of LITA-LAD rather than on graft count or conduit hierarchy. From this perspective, the central task is not to pursue increasing conduit complexity for its own sake, but to build a reliable surgical strategy around a durable, high-quality LITA-LAD.

### 8.2. Why LITA-LAD Can Sustain the Prognostic Value of CABG

The practical resolution of the second-graft controversy, with respect to survival superiority, suggests that the survival benefit of CABG appears to be driven primarily by LITA-LAD, while the additional prognostic effect of a second graft to a non-LAD territory is difficult to demonstrate. This interpretation can be understood in relation to the intrinsic flow-supplying capacity of the LITA and the LAD-centered structure of myocardial perfusion. As discussed in Section 3, the LITA can provide a large and adaptive blood supply to ischemic organs [11]. The LAD also occupies a unique anatomical and physiological position. It supplies the anterior wall and interventricular septum directly, while its diagonal branches may contribute to the lateral wall and its septal branches may support broader collateral flow toward the inferior wall and, in selected circumstances, the right ventricular myocardium. Thus, LITA-LAD should not be viewed merely as a graft to one coronary branch. It may function as a central inflow route into a wider left ventricular perfusion system.

From this perspective, ischemic events in non-LAD territories may not depend solely on the second graft. If a new lesion develops in a non-LAD vessel, or if a second graft gradually loses part of its flow-supplying capacity, the LITA-LAD pathway may still provide compensatory perfusion through the LAD-centered network. Conversely, when the second graft maintains adequate flow to its target territory, the compensatory contribution from LITA-LAD is less prominent. This may help explain why differences in second-graft selection have not translated into a clearly detectable survival advantage in randomized trials. The mechanism is difficult to prove directly, because it involves time-dependent changes in graft flow, native coronary disease progression, collateral development, and regional myocardial demand. Nevertheless, within this hypothesis-generating physiological framework, the results of the second-graft controversy can be interpreted as suggesting that LITA-LAD provides the principal prognostic component of CABG, while additional grafts contribute mainly by completing or supporting myocardial flow rather than by independently determining long-term survival.

### 8.3. Maximizing the Advantages of the LITA

Although arterial grafts are often treated as a single category, the ITA has distinctive histological advantages, and the LITA has additional anatomical advantages. Like the aorta, the ITA is an elastic artery, characterized by minimal atherosclerotic plaque formation and sparse vasospasm-related innervation [74]. In addition, because of the left-sided position of the heart, the LITA lies closer to the LAD than the RITA and permits a shorter and more direct in situ graft course. These advantages can be further enhanced by meticulous harvesting. Skeletonized LITA harvesting can provide greater usable graft length and may better preserve parasternal collateral circulation than pedicled harvesting, thereby helping to maximize the reach and safety of the conduit [75,76]. The CABG strategy should therefore emphasize optimal use of this anatomically and biologically favorable conduit.

### 8.4. Beyond the Symbol of LITA-LAD: The Technical Substance of the Foundational Anastomosis

Although LITA-LAD is widely regarded as the foundational anastomosis of CABG, its technical substance has rarely been described in a sufficiently concrete manner. Even when surgeons seek to learn or refine the actual method of constructing a high-quality LITA-LAD anastomosis, the available literature often provides only general descriptions rather than explicit technical guidance. This may reflect the long-standing symbolic treatment of LITA-LAD: it has often been recorded as present or absent, while the operative quality of its construction has remained difficult to evaluate. However, because LITA-LAD forms the central anastomosis of CABG, its construction should not remain an implicit technical assumption.

**Figure 2:**
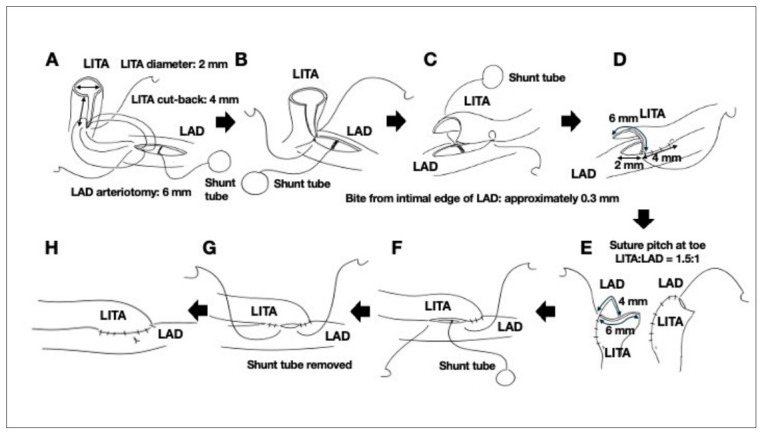
Stepwise technical schema of LITA-LAD anastomosis using a parachute suturing technique and an intracoronary shunt. (**A**) Initial parachute suturing after preparation of the LITA and LAD. (**B**) Appearance after the parachute sutures have been lowered. (**C**) Placement of the first side stitch as viewed from the assistant’s side. (**D**) Completion of the assistant-side suture line, leaving approximately 2 mm of the LAD arteriotomy toward the toe. (**E**) Superior view of toe suturing. At the toe, the suture pitch is adjusted to an approximate LITA-to-LAD ratio of 1.5:1, creating a cobra-head-like anastomotic configuration. (**F**) Suturing of the surgeon-side suture line. (**G**) Removal of the intracoronary shunt tube before completion of the anastomosis. (**H**) Completed LITA-LAD anastomosis. Arrows indicate the direction of the procedural sequence shown in each panel. The dimensions shown represent a typical technical schema rather than a fixed rule and should be adjusted according to the size, quality, and orientation of the LITA and the LAD. A distinctive feature of this technique is that, on the LAD side, each stitch is consistently passed from the intimal side to the adventitial side. This technical schema is intended to illustrate that LITA-LAD quality is technically dependent and should not be reduced to a binary procedural variable.

**Figure 3:**
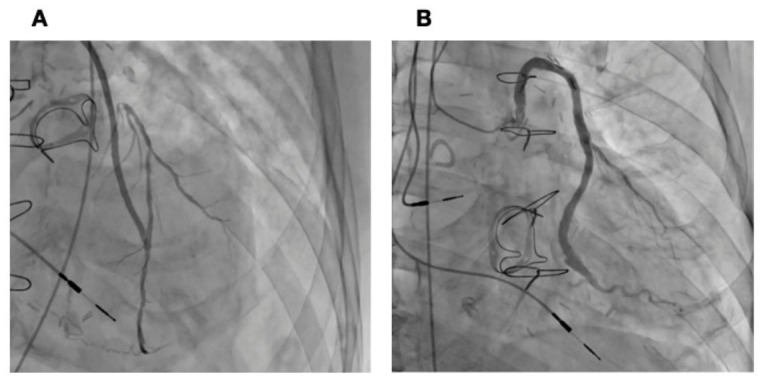
Representative 10-year postoperative angiographic images of LITA-LAD and GSV sequential grafts. (**A**) Angiography of the LITA-LAD graft 10 years after CABG demonstrates preserved graft patency and antegrade flow to the LAD territory. (**B**) Angiography of the GSV sequential graft 10 years after CABG demonstrates preserved patency of the sequential conduit and preserved distal runoff to the target coronary branches. These images illustrate the long-term durability of high-quality LITA-LAD reconstruction and the complementary role of GSV sequential grafting for non-LAD territories. GSV, great saphenous vein; LAD, left anterior descending artery; LITA, left internal thoracic artery. These angiographic images are illustrative examples from the authors’ practice and are not intended to demonstrate generalizability.

At its core, the value of LITA-LAD lies in establishing stable flow from the LITA to the LAD. In this sense, the essential requirement is simple: the LITA must be appropriately anastomosed to the LAD and must function reliably. Nevertheless, because the quality and reproducibility of this anastomosis are clinically important, technical optimization remains meaningful. Figure 2 therefore illustrates one example of a structured approach to LITA-LAD construction based on the first author’s practice. This figure is presented not as a standardized or exclusive method, but as a concrete attempt to make explicit the technical principles that may contribute to a stable and high-quality LITA-LAD. The functional performance of this LITA-LAD strategy has been examined in relation to the first author’s empirical intraoperative graft-flow study describing the total graft flow plateau (TGFP) [77], and its long-term anatomical durability is further illustrated by 10-year postoperative angiography (Figure 3A). The on-lay patch technique also retains value in selected patients with diffuse coronary disease, as it can expand the applicability of CABG without requiring more aggressive coronary reconstruction [78,79].

Rare situations may arise in which the LITA cannot be used or is damaged during harvesting. This issue should be distinguished from the second-graft controversy, because it concerns preservation of the primary LAD-centered inflow itself. In such circumstances, the operative priority is not to pursue a predefined conduit hierarchy, but to secure a durable and reliable inflow to the LAD. The RITA represents a direct substitute, because it shares the biological properties of the LITA and has shown excellent long-term patency when grafted to the LAD [80]. When RITA use requires bilateral ITA harvesting, the risk of sternal wound complications should be considered; however, data from ART suggest that skeletonized bilateral ITA harvesting may mitigate this risk compared with pedicled bilateral ITA harvesting [81]. A no-touch GSV may also be a practical alternative, particularly when bilateral ITA harvesting is undesirable or the RITA is unsuitable, because no-touch GSV grafting to the LAD has shown favorable patency and has been proposed as a substitute for the LITA in elderly or high-risk patients [82]. Thus, when LITA-LAD cannot be achieved, the essential principle remains unchanged: a durable LAD-centered inflow should be secured by the best available conduit according to patient risk, conduit quality, and target-vessel anatomy.

### 8.5. Surgical Access Should Serve LITA-LAD Quality

Median sternotomy allows extensive harvesting of the LITA and reliable construction of the LITA-LAD anastomosis. Choosing a smaller incision may be reasonable in selected patients, but it should not compromise the quality of LITA-LAD, which remains a major technical advantage of CABG. This concern is not merely theoretical: early angiographic studies of minimally invasive direct coronary bypass reported anastomotic stenosis, graft occlusion, and the need for reintervention, emphasizing that limited-access coronary surgery should ultimately be evaluated according to the quality and patency of the LITA-LAD anastomosis [83,84].

### 8.6. Choosing the Operative Strategy According to Technical Reliability

In addition, various adjunctive technologies are now available to support beating-heart coronary anastomosis. The choice among off-pump coronary artery bypass (OPCAB), beating-heart CABG with CPB or other mechanical circulatory support, and standard CABG should therefore be guided by the surgeon’s capability and the patient’s condition. Although OPCAB has often been viewed critically based on studies reporting inferior graft patency, fewer completed grafts, or worse long-term outcomes [85,86], these findings should be interpreted in relation to technical reproducibility and case selection. Beating-heart CABG also offers important practical advantages: graft length can be adjusted more precisely, flow can be assessed immediately with transit-time flow measurement (TTFM) after completion of the anastomosis, and if necessary, revision can be performed without delay.

### 8.7. Reconsidering Complete Revascularization: From Graft Count to Physiological Flow Distribution

Coronary angiography is indispensable for selecting target vessels in CABG. However, its visual clarity may also create a conceptual limitation. By displaying coronary arteries as separate anatomical territories, angiography may encourage an anatomical view in which each visible stenosis is assumed to require a corresponding graft. In this framework, complete revascularization is easily equated with increasing the number of distal anastomoses. This lesion-oriented concept also resembles conventional angiography-guided PCI, later challenged by the FAME trial series [71].

However, this anatomical interpretation does not fully explain the physiology of CABG. In many patients, total graft flow reaches a plateau after approximately three grafted targets, and additional anastomoses do not further increase total graft flow. Instead, flow per graft begins to decrease [77]. This empirical intraoperative graft-flow finding, referred to as TGFP, suggests that angiographically visible territories are not physiologically independent compartments. Rather, they may interact through a mutually compensatory perfusion system. Although TGFP was derived from empirical graft-flow measurements, its broader physiological interpretation remains hypothesis-generating and requires external validation. Excessive grafting may therefore increase the risk of graft competition.

The observation of TGFP supports a cautious reconsideration of the traditional concept of complete revascularization. Complete revascularization should not mean grafting as many stenotic lesions as possible; rather, it should mean satisfying myocardial flow demand through carefully selected target vessels. In this sense, CABG strategy should shift from an anatomical “more is better” approach to a physiological “targeted flow sufficiency” approach. The goal is not to maximize the number of anastomoses, but to achieve sufficient flow distribution through carefully selected major perfusion routes.

A useful reference point is resting myocardial blood flow demand. Although it varies according to body size, ventricular mass, myocardial hypertrophy, severity of ischemia, and native coronary flow, the resting blood flow demand of normal myocardium is approximately 250 mL/min [87]. In CABG, total graft flow has been reported to reach a plateau at approximately 160 mL/min [77]. These values should not be interpreted as rigid thresholds. Intraoperative graft flow represents only the surgically added component of myocardial perfusion, because native coronary flow usually remains present to varying degrees. Future CABG strategy may therefore be guided not only by angiographic anatomy, but also by expected myocardial flow demand and intraoperative confirmation of total graft flow using TTFM.

From this perspective, CABG can be understood as a flow-distribution system whose success depends on selecting appropriate inflow routes, achieving sufficient total graft flow up to the plateau, and avoiding unnecessary grafting beyond the point at which additional anastomoses are unlikely to increase effective perfusion.

### 8.8. Pragmatic Second-Graft Selection: The No-Touch GSV and Complementary Techniques

From the perspective of balancing low procedural burden with high long-term patency, the no-touch GSV technique, introduced by Souza and colleagues, appears to offer a practical approach to the second-graft controversy [88]. Before turning to the no-touch GSV graft, the limitations of arterial conduits other than the LITA should be briefly considered. As discussed above, the RITA is anatomically less favorable and, when combined with the LITA, may increase the incidence of mediastinitis, especially in diabetic patients [38,89]. Other candidate arterial conduits are muscular arteries and therefore more susceptible to vasospasm. The RA was previously abandoned as a graft because of unsatisfactory early results [31,32]. Its use may also compromise future dialysis access in patients with severe renal dysfunction [90], and previous trans-radial catheter intervention raises concerns about graft quality because of intimal thickening [91]. RGEA may interfere with future abdominal surgical procedures [92,93], and its long-term patency has been less consistent than that of the ITA [94].

The favorable long-term patency of the no-touch GSV has been demonstrated in randomized long-term angiographic follow-up studies [95,96]. Although harvest-related wound complications have been reported, they can be mitigated to some extent by meticulous technique, and the overall long-term performance is sufficiently favorable to support its use as a second graft. In addition, sequential bypass remains a practical strategy, allowing broad myocardial coverage with a limited number of conduits while reducing the number of proximal anastomoses [97,98]. The long-term anatomical durability of this strategy is further illustrated by 10-year postoperative angiography (Figure 3B).

### 8.9. Consolidating a Practical Contemporary CABG Strategy

The basic framework described in this chapter had already been established by the 1980s, although it has since been refined by techniques such as no-touch GSV harvesting. The contemporary evidence reviewed here suggests that CABG strategy can now be consolidated around high-quality LITA-LAD construction, physiologically sufficient grafting, and pragmatic complementary conduit selection. Cardiac surgeons should continue to refine LITA-LAD technique, master complementary grafting techniques such as no-touch GSV harvesting, sequential bypass, and on-lay patch reconstruction, and maintain consistently stable surgical outcomes. In this framework, the enduring value of CABG lies not in increasing procedural complexity, but in reproducibly delivering durable LAD-centered revascularization with appropriate support for non-LAD territories.

## 9. Future Perspectives

### 9.1. Reframing Future Progress Around LITA-LAD Quality

In valvular and aortic disease, therapeutic progress has often been driven by advances in prosthetic materials and devices, which have reshaped surgical strategy itself. In ischemic heart disease, however, the course has been somewhat different. Even PCI, despite rapid and continuous technological innovation, did not establish the central prognostic role that many had anticipated. Instead, the long arc of clinical evidence has pointed back to the enduring importance of LITA-LAD bypass. Future progress in this field may therefore not lie primarily in further device development or advances in molecular biology. Rather, an important remaining frontier may be the refinement of clinical assumptions and surgical priorities. A practical change in mindset is needed: LITA-LAD should be recognized as the essential component of surgical revascularization whose quality should be maximized. At the same time, second and subsequent grafts should be selected with greater emphasis on minimizing patient burden while providing adequate non-LAD perfusion. These priorities do not depend on future breakthroughs. They are already before us and require consistent recognition, technical refinement, and implementation in daily surgical practice.

### 9.2. Interpreting Forthcoming Evidence Within the LITA-LAD Framework

Forthcoming or ongoing trials, including ROMA and contemporary comparisons of PCI and CABG in ischemic left ventricular dysfunction, may provide additional evidence relevant to the issues discussed in this review [99,100]. However, these studies can be interpreted within a LITA-LAD-centered framework. In trials of multiple arterial grafting, the key question is whether additional arterial conduits provide a measurable survival benefit on a shared LITA-LAD background, in addition to any effects on selected components of composite endpoints. Similarly, future comparisons among PCI, CABG, and medical therapy should clarify which patients derive prognostic benefit from LITA-LAD-based surgical revascularization in addition to contemporary medical therapy. Future studies should therefore evaluate the quality, patency, and functional contribution of LITA-LAD, the physiological adequacy of total graft flow, and long-term survival, rather than relying primarily on procedural categories or broad composite endpoints.

## 10. Conclusions

The 150-year history of ischemic heart disease treatment can be viewed, in essence, as a century dominated by nitrates, followed by an era in which LITA-LAD has remained a central surgical strategy to this day. Many treatments and concepts have risen and faded, but beneath much of this apparent progress lay a recurrent underappreciation of LITA-LAD.

In the past quarter century, the field was particularly unsettled by the prolonged second-graft controversy and by repeated comparisons with PCI. Nevertheless, cardiac surgeons throughout the world continued to treat patients successfully with LITA-LAD and GSV grafting to the non-LAD territories. In retrospect, this was a practical and durable strategy.

Alongside evidence-based medical therapy, LITA-LAD remains central to the ability of CABG to improve long-term survival. This anatomical and physiological advantage should be preserved through meticulous technique and appropriate patient-centered graft selection. Our responsibility is to preserve this advantage, reproduce LITA-LAD reliably, and transmit its technical principles to the next generation.

We hope that younger generations of cardiac surgeons and cardiologists will study this history carefully and will develop the critical perspective needed to recognize evidence-supported principles and to distinguish them from assumptions that require continued reappraisal.

## Figures and Tables

**Table 1:** Representative randomized evidence comparing coronary bypass conduits.

Study	Country	Study Design	Published Year	Number of Patients	Index LAD Graft	Second Graft to Non-LAD	Key Hard Endpoint	Longest Follow-Up
Zeff [22]	US	RCT	1988	80	ITA or GSV	GSV	Survival	10 years
RSVP [41]	UK	RCT	2008	142	ITA	RA or GSV	Patency	5 years
Goldman CSP 474 2011 [36]	US	RCT	2011	757	ITA	RA or GSV	Patency	1 year
ART [38]	UK	RCT	2016	3102	ITA	ITA or GSV	Survival	5 years
RADIAL [42]	International	RCT, Meta-analysis	2018	1036	ITA	RA or GSV	Composite events ^†^	12 years
ART [39]	UK	RCT	2019	3102	ITA	ITA or GSV	Survival	10 years
RADIAL [43]	International	RCT, Meta-analysis	2020	1036	ITA	RA or GSV	Composite events ^†^	15 years
RAPCO-RITA [44]	Australia	RCT	2020	394	ITA	ITA or RA	Survival	10 years
RAPCO-SV [44]	Australia	RCT	2020	225	ITA	RA or GSV	Survival	10 years
Goldman CSP 474 2022 [37]	US	RCT	2022	726	ITA	RA or GSV	Survival	18 years
RAPCO [45]	Australia	RCT	2023	394 + 225	ITA	ITA or RA ITA or GSV	Survival	15 years

GSV, great saphenous vein; ITA, internal thoracic artery; LAD, left anterior descending artery; RA, radial artery; RCT, randomized controlled trial. ^†^ In RADIAL, the principal outcome was a composite of death, myocardial infarction, and repeat revascularization rather than all-cause survival alone.

**Table 2:** Major randomized trials involving PCI, CABG, and medical therapy and selected conditions expected to favor prognostic separation.

Study	Country	Year	No. of Patients	Low-EF ICM	Complex 3VD	High-Risk LM Disease	Adequate DM Inclusion	≥10 Years Follow-Up	Death/MI as a Key Endpoint	No. of Selected Criteria Met *n*/6
PCI versus CABG										
EAST [50]	US	1994	392	No	Limited	No	Limited	No	Limited	0/6
BARI [51,52]	US	1996 2007	1829	No	Limited	No	Limited	Yes	Yes	2/6
SoS [53]	Europe Canada	2002	988	No	Limited	No	Limited	No	Limited	0/6
MASS II [54]	Brazil	2004	611	No	Limited	No	Limited	Yes	Limited	1/6
SYNTAX [55,56] SYNTAXES [57]	International	2009	1800	Limited	Yes	Limited	Limited	Yes	Limited	2/6
PRECOMBAT [58]	South Korea	2011	600	No	Limited	Limited	Yes	Yes	Limited	2/6
FREEDOM [59]	US	2012	1900	No	Limited	No	Yes	No	Yes	2/6
BEST [60]	South Korea	2015	880	No	Limited	No	Yes	Yes	Limited	2/6
EXCEL [61]	US	2016	1905	No	Limited	Limited	Yes	No	Limited	1/6
NOBLE [62,63,64]	North Europe	2016 2020 2026	1184	No	Limited	Limited	Limited	No	Limited	0/6
PCI + Medical Therapy versus Medical Therapy										
RITA-2 [65,66]	UK	1997 2003	1018	No	No	No	No	No	Limited	0/6
COURAGE [67]	US Canada	2007	2287	No	Limited	No	Yes	Limited	Yes	2/6
REVIVED-BCIS2 [68]	UK	2022	700	Yes	Limited	Limited	Yes	No	Yes	3/6
CABG + Medical Therapy versus Medical Therapy										
STICH [69] STICHES [70]	International	2011 2016	1212	Yes	Yes	No	Yes	Yes	Yes	5/6

CABG, coronary artery bypass grafting; DM, diabetes mellitus; EF, ejection fraction; ICM, ischemic cardiomyopathy; LM, left main; MI, myocardial infarction; PCI, percutaneous coronary intervention; 3VD, three-vessel disease. Low-EF ICM was defined as ischemic cardiomyopathy with markedly reduced LVEF, approximately ≤35%. DM was rated as Yes when diabetic patients accounted for ≥30% of the study population. Death/MI was rated as Yes when all-cause death, cardiovascular death, or spontaneous myocardial infarction was a central endpoint. Ratings were assigned as follows: Yes, clear fulfillment; Limited, underrepresentation, clinical or anatomical selection, or limited centrality; No, absence or exclusion. For high-risk LM disease, Limited denotes selected PCI-feasible LM lesions rather than broader high-risk LM anatomy.

**Table 3:** PCI technology, CABG strategy, funding category, and main outcome summary in major randomized trials involving PCI, CABG, and medical therapy.

Study	Country	Year	PCI Technology	CABG Strategy	Funding Category	Main Outcome Direction
PCI versus CABG						
EAST [50]	US	1994	PTCA	LITA-LAD-based	Public/academic funding	Neutral overall
BARI [51,52]	US	1996 2007	PTCA	LITA-LAD-based	Public funding	Neutral overall CABG favored in diabetes
SoS [53]	Europe Canada	2002	BMS	LITA-LAD-based	Industry support	CABG favored
MASS II [54]	Brazil	2004	BMS	LITA-LAD-based	Public/academic funding	CABG favored
SYNTAX [55,56] SYNTAXES [57]	International	2009 2013 2019	First- generation DES	LITA-LAD-based	Industry support	CABG favored in complex disease/3VD 10-year mortality neutral
PRECOMBAT [58]	South Korea	2011	First- generation DES	LITA-LAD-based	Public/academic funding with industry support	Neutral overall
FREEDOM [59]	US	2012	First- generation DES- dominant	LITA-LAD-based	Public/academic funding with industry support	CABG favored
BEST [60]	South Korea	2015	Second- generation DES	LITA-LAD-based	Public/academic funding with industry support	CABG favored
EXCEL [61]	US	2016	Second- generation DES	LITA-LAD-based	Industry support	Neutral primary endpoint
NOBLE [62,63,64]	North Europe	2016 2020 2026	Mixed/ newer- generation DES	LITA-LAD-based	Public/academic funding with industry support	CABG favored
PCI + Medical Therapy versus Medical Therapy						
RITA-2 [65,66]	UK	1997 2003	PTCA	-	Public funding	Medical therapy equivalent
COURAGE [67]	US Canada	2007	BMS- dominant	-	Public funding	Medical therapy equivalent
REVIVED-BCIS2 [68]	UK	2022	Newer- generation DES	-	Public funding	Medical therapy equivalent
CABG + Medical Therapy versus Medical Therapy						
STICH [69] STICHES [70]	International	2011 2016	-	LITA-LAD-based	Public funding	CABG + medical therapy favored

BMS, bare-metal stent; CABG, coronary artery bypass grafting; DES, drug-eluting stent; LITA-LAD, left internal thoracic artery to left anterior descending artery; PCI, percutaneous coronary intervention. PCI technology was summarized by major device generation rather than by individual stent platforms; PTCA, percutaneous transluminal coronary angioplasty. First-generation DES mainly included sirolimus- and paclitaxel-eluting stents, whereas second-generation or newer-generation DES included everolimus-, zotarolimus-, and biolimus-eluting stents. Specific stent platforms and drug types are described in the original trial publications. CABG strategy was summarized as LITA-LAD-based CABG with additional grafting to non-LAD territories, representing the common basic structure across trials.

## Data Availability

The original contributions presented in this study are included in the article. Further inquiries can be directed to the corresponding author.

## Abbreviations

The following abbreviations are used in this manuscript:

ACS	Acute coronary syndrome
BMS	Bare metal stent
BITA	Bilateral internal thoracic artery
CABG	Coronary artery bypass grafting
CAG	Coronary angiography
CPB	Cardiopulmonary bypass
DES	Drug-eluting stent
ECG	Electrocardiogram
GSV	Great saphenous vein
ITA	Internal thoracic artery
LAD	Left anterior descending artery
LCx	Left circumflex artery
LITA	Left internal thoracic artery
OPCAB	Off-pump coronary artery bypass
PCI	Percutaneous coronary intervention
RA	Radial artery
RCA	Right coronary artery
RCT	Randomized controlled trial
RGEA	Right gastroepiploic artery
RITA	Right internal thoracic artery
SITA	Single internal thoracic artery
TGFP	Total graft flow plateau
TTFM	Transit-time flow measurement
